# Reporting clinical trials of psychosocial interventions in child and adolescent psychiatry and mental health

**DOI:** 10.1186/1753-2000-5-4

**Published:** 2011-02-28

**Authors:** Lutz Goldbeck, Benedetto Vitiello

**Affiliations:** 1 Department of Child and Adolescent Psychiatry/Psychotherapy, University Hospital Ulm, Steinhövelst 5, 89075 Ulm, Germany; 2 Child and Adolescent Treatment and Preventive Intervention Research Branch, National Institute of Mental Health, Bethesda, Maryland, USA

## Editorial

Randomized Clinical Trials (RCTs) are a powerful, although not the only, way of scientifically investigating treatment efficacy and effectiveness. As early as 1753, the Scottish physician James Lind used this method to test the effect of citric acid in the prevention of scurvy when he randomly assigned sailors to six treatment arms. By the middle of the 20^th ^century, RCTs had become the gold standard of evidence based medicine. The first published RTC demonstrated the efficacy of streptomycin in tuberculosis, even though under double-blind conditions or with a placebo control condition [1].

Compared with pharmacology research, the application of the RCT to psychotherapy research has taken time to be widely adopted. In 1990, concordant with the emerging paradigm of Evidence Based Medicine (EBM), the American Psychological Association developed criteria for considering psychological treatments as evidence-based, and recommended either RCT group designs or a large series of single case experiments, together with the development of treatment manuals, a clear specification of trial participants, and the need to replicate a finding of efficacy [2].

A number of criticisms of this position have been put forward, based on issues such as the difficulty of designing a placebo-like control, the impossibility of maintaining double-blind conditions, the challenge of standardizing interventions, the importance of the therapist-patient fit, and the reduced external validity of psychotherapy in the context of a RCT [3]. In spite of these limitations, since no valid alternatives currently exist for producing generalizable evidence, the RCT is now accepted as the best way of proving efficacy also in psychotherapy.

The value of a RCT can be realized only when it has been reported clearly and comprehensively. Detailed criteria for determining the quality of a psychotherapy RCT have been developed [4]. Clinicians need to appreciate the validity of any published trial before they may decide to implement a treatment in their clinical practice. Other researchers should be able to fully understand that critical elements of each RCT in order to plan further research or conduct meta-analyses.

In the past two decades, the Consolidated Standards of Reporting Trials (CONSORT) were developed to improve clinical trial reports (for the recent revision see [5]. A CONSORT checklist is provided and the use of a flow diagram of progress through stages of a RCT is highly recommended (see http://www.consort.org). These standards have also impacted the study design of new trials, especially since most clinical journals adopted the CONSORT statement not only to guide authors in providing comprehensive information on all the important details of their trial, but also to make decisions about publishing the study. Reports of less rigorous studies are probably to be rejected, as they may contribute only inconclusive results to the literature.

As a result of the broad discussion of the appropriateness of the CONSORT statement for psychosocial and other non-pharmacological interventions, additional criteria for reporting have been developed [6,7], such as eligibility criteria for centers and therapists, details of the randomization procedure, the number of therapists and centers per condition, the number of patients treated by one therapist, detailed description of both the experimental treatment and comparator(s) and of its implementation, description of the different components of the interventions and of individually tailored application of these components, information on addressing clustering effects, allocation of therapists to conditions, blinding of participants and evaluators to group assignment, and finally considering the choice of comparator, lack of or only partial blinding, and unequal expertise of therapists and centers involved in the trial. It is obvious that inclusion of this information improves the interpretation of trial results by the reader.

Reviews and meta-analyses of trial reports of psychological interventions often reveal insufficient and incomplete information [8]. This is not only due to a neglect of the CONSORT statement and its extensions by the authors of the primary studies, but may also be due to the often restrictive word limits in print journals. Many trial reports fail to provide clear-cut evidence of treatment effect because of methodological shortcomings, rather than intrinsic inefficacy of the treatment. It is therefore imperative to clearly identify the design and methodological characteristics of reported RCT so that the readers can fully appreciate the strengths and limitations of these experiments.

Since its founding in 2007, *Child and Adolescent Psychiatry and Mental Health *has adopted the CONSORT statement and all authors are strongly recommended to use the resources of the CONSORT website. As word limits are not a problem in an online journal, there is sufficient room for providing all necessary information in a detailed manner.

The study of Melfsen et al. [9] on the effectiveness of a cognitive behavioral therapy of children with social phobia is an example for a study with both significant strengths and important limitations, such as a small sample size. However, by providing a thorough description of the study design and methods and including a CONSORT of the study sample, the authors allow the reader to fully appreciate what the study can and cannot provide in term of evidence, thus enhancing the value of the data.
